# The Effect of a Small Amount of Water on the Structure and Electrochemical Properties of Solid-State Synthesized Polyaniline

**DOI:** 10.3390/ma5101811

**Published:** 2012-10-10

**Authors:** Weiwei Shao, Ruxangul Jamal, Feng Xu, Aminam Ubul, Tursun Abdiryim

**Affiliations:** Key Laboratory of Petroleum and Gas Fine Chemicals, Educational Ministry of China, College of Chemistry and Chemical Engineering, Xinjiang University, Urumqi 830046, China; E-Mails: 693680739@163.com (W.S.); jruxangul@xju.edu.cn (R.J.); 202xufeng@163.com (F.X.); aminam520@gmail.com (A.M.)

**Keywords:** conducting polymers, solid-state polymerization, structure-property relations, polyaniline

## Abstract

A series of polyaniline (PANI) salts were synthesized with the presence of a small amount of water varying from 0 to 1 mL at the beginning of solid-state polymerization. The structure and morphology of the samples were characterized by fourier transform infrared (FTIR) spectra, ultraviolet-visible (UV-Vis) absorption spectra, X-ray diffraction (XRD) and transmission electron microscopy (TEM). The electrochemical performances of the products were investigated by galvanostatic charge-discharge, cyclic voltammetry, cycling stability and electrochemical impedance spectroscopy (EIS). The results showed that the amounts of water can affect the oxidation degree, conjugate level and crystallinity of PANI salts. All PANI salts showed spherical morphology with the diameter of about 60 nm as shown by TEM. The electrochemical tests showed the highest specific capacitance value 593.3 F^.^g^−1^ in 1 M H_2_SO_4_ for PANI prepared with the addition of 0.5 mL of water at the beginning of solid-state polymerization.

## 1. Introduction

Conducting polyaniline (PANI), composed of benzenoid and quinonoid units with the delocalized conjugated structures, has several redox states, which has become the research focus in the field of conducting polymers [1,2,3,4,5,6,7,8,9]. The conductivity and processability of PANI can be adjusted through the selection of a suitable dopant and the varying of the oxidation states [10,11]. Generally, the organic sulfonic acids are widely used as doping agents, which can improve the solubility and electrochemical performances of PANI [12,13]. PANI can be synthesized electrochemically or chemically by oxidative polymerization of aniline [14,15]. With the electrochemical method, it is easy to control the morphology and electrical properties of PANI, but its disadvantage lies in mass production [16]. On the contrary, the chemical method is considered to be more effective for commercial mass production.

Recently, the solid-state synthesis has aroused researchers’ attention because a small amount of solvent or no solvent is used in the reaction system, and the reactants are brought into intimate contact by grinding or through the ball-milling process. Due to low costs, reduced pollution, and simplicity in process and handling, the solid-state synthesis can be used for a large scale production [17]. Now, solid-state synthesis is widely applied to prepare the PANI-type conducting polymers [18,19,20,21,22,23]. In the solid-state synthesis method, the reaction chiefly occurs on the surface of a solid-sate reactant and the inter-diffusion rate of the reactants is much slower than that of a traditional solution method [18]. This means that by carefully controlling the experimental conditions to adjust the nucleation and growth of the polymer, the solid-state polymerization method could be used to fabricate the nanostructured polymer [19,20,21,22,23]. Huang *et al.* report a solvent-free mechanochemical route to PANI in which the reaction is induced by ball-milling the solvent-free anilinium salt and oxidant under ambient conditions [18]. Our group has successfully synthesized PANI and its derivatives doped with different acids with the solid-state polymerization method, in which the small amount of water is added at the beginning of the reactions of aniline and doping acid to form the anilinium salt, and the residual moisture in the reaction system can be gradually evaporated after the adding of the oxidant and the reaction process [19,24,25]. Moreover, we have found that the presence of a small amount of water at the beginning of the reactions can take on the role of catalyst, which can accelerate the reaction rate and increase the doping degree of the acids. However, until now there has been no systematic information concerning the effect of a small amount water on the structure and properties of the PANI.

Herein, we report the preparation of polyaniline doped by *p*-TSA with the presence of a small amount of water varying from zero to one mL in the beginning of the solid-state reaction. The influence of the small amount of water on the structure and electrochemical properties of PANI was deeply discussed based on the results from fourier transform infrared (FTIR) spectra, ultraviolet-visible (UV-Vis) absorption spectra, X-ray diffraction (XRD), transmission electron microscopy (TEM),, galvanostatic charge-discharge, cyclic voltammetry, cycling stability and electrochemical impedance spectroscopy (EIS).

## 2. Results and Discussion

### 2.1. Fourier Transform Infrared (FT-IR) Spectra

**Figure 1:**
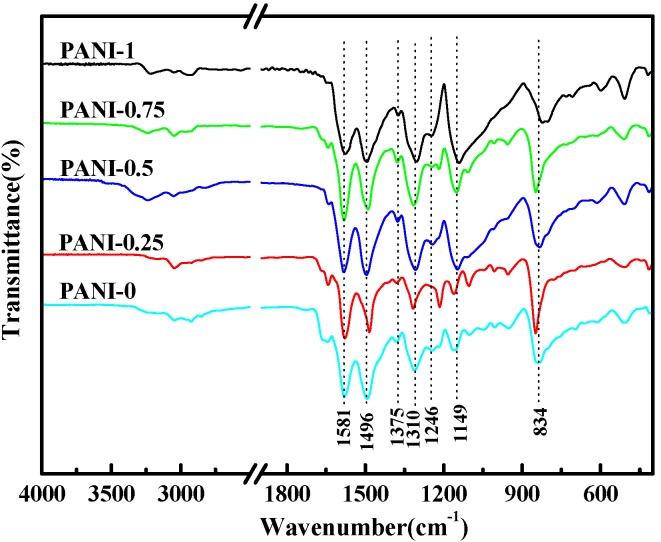
Fourier transform infrared (FTIR) spectra of Conducting polyaniline (PANI) with different amounts of water.

Figure 1 shows the FTIR spectra of PANI salts synthesized by the solid-state synthesis method. The main characteristic peak positions of all PANI salts are almost same, the broad band at ~3205 cm^−1^–3321 cm^−1^ is attributable to the N–H stretching vibration due to the protonation of nitrogen [10,11], ~3050–3109 cm^−1^ is assigned to stretching vibration of the aromatic C-H bond and the characteristic band at ~2835–2949 cm^−1^ can be due to the stretching vibration of the methyl group (-CH_3_). The two bands appearing at ~1565–1578 cm^−1^ and ~1487–1497 cm^−1^ correspond to the stretching vibration of quinoid and benzenoid ring, respectively. The peak at ~1375cm^−1^ is attributed to the C–N= stretching vibration between benzenoid and quinoid units [26]. The band at ~1297–1311 cm^−1^ can be assigned to the π-electron delocalization induced in the polymer through protonation or C-N-C stretching vibration, while the peak at ~1246 cm^−1^ is due to the C-N^+^ stretching vibration in the polaron structure [19,24]. The band at 1145–1161cm^−1^ is assigned to the plane bending vibration of C–H (modes of N=Q=N, Q=NH^+^–B and B–NH^+^–B, Q represents the quinoid ring and B represents the benzenoid ring), which is formed during protonation [27]. It is described as the “electronic-like band” and considered to be a measure of the degree of delocalization of electrons of PANI [14]. The peak at ~1105 cm^−1^ is attributed to the aromatic C-H bending in the plane for the 1,4-disubstituted aromatic ring [28] and the band appearing at ~830 cm^−1^ is attributed to an aromatic C-H out-of-plane bending vibration [29]. The peaks at ~1050 cm^−1^ and ~690 cm^−1^ are relative to the S=O and S–O stretching vibration of the sulphonate groups attached to the aromatic rings, which also indicates that the prepared PANI nanostructures were in the doping state [30] The presence of characteristic bands confirms that all PANI salts contain the conducting emeraldine salt phase [18,30]. The differences of intensities from 1350–1000 cm^−1^ between PANI salts may be due to the differences in the protonation level and oxidation level or to the change in stability of the conjugated system [31].

The oxidation degree of the PANI can be determined by the relative intensities ratio of peak corresponding to the quinoid to benzenoid ring modes [25,32]. Calculating the relative intensity ratio (*I*_~1565–1570_/*I*_~1487–1497_) of all PANI salts, the specific values are 0.96, 1.10, 0.97, 1.18, 0.86 in the order of PANI-0, PANI-0.25, PANI-0.5, PANI-0.75, PANI-1, respectively. These results above indicate that the oxidation state can be affected by a small amount of water; this may be related to the amount of H^+^ and the oxidant in the reaction medium [33]. As for PANI-0, there is no water in the reaction system. However, the liquid aniline can react with *p*-TSA. After adding APS, the contact probability of the oxidant is much less due to no water, thus facilitating the formation of the reduction state of polymer. When adding 0.25 mL of H_2_O, the too little amount of H^+^ and the raised contact probability between APS and aniline salt facilitates the formation of the oxidation state [33]. However, the amount of H^+^ is increased by adding 0.5 mL of H_2_O, and more H^+^ is inclined to the formation of the reduction state even thoughthe oxidant facilitates to form the oxidation state. Considering the two impacts, the ratio (*I*_~1565–1570_/*I*_~1487–1497_) of PANI-0.5 is 0.97, which is close to 1. It bears a dark green color at the end of the experiment, indicating further that the formation of polyaniline is in its doped emeraldine oxidation state [32]. For PANI-0.75, on the other hand, the amount of H^+^ and the contact probability of the oxidant are both higher, so more oxidant tends to initiate aniline to produce the oxidation state. When adding 1 mL of H_2_O, the amount of H^+^ will be increased by increasing the soluble amount of *p*-TSA with the presence of more water. This will cause the separation of the oxidant from the monomer, which, in turn, facilitates the formation of the reduction state of polymer during the oxidative polymerization. This means the oxidation degree of PANI-1 should be lower. It is well known that the reactants are brought into intimate contact through the grinding process, and inter-diffusion of the reactants is necessary for the solid-state synthesis. It should be noted that this solid-state synthesis in the beginning involves solid and liquid phases, *i.e.*, the solid monomer salt and a small amount of water (liquid aniline and *p*-TSA were partly grinded to form solid aniline salt). Because the oxidative polymerization is exothermic, after adding APS oxidant, the reaction only happens in a solid state. With the presence of more water, the reaction medium will be more acidic, because the the amount of H^+^ will be increased by increasing the soluble amount of *p*-TSA with more water. The small portion of aniline salts in the liquid phase will increase the inter-diffusion rate of the reactants and probability of reaction. Therefore, the more water added in the reaction, the more yield of the corresponding product there will be, as can been seen in Table 1.

**Table 1:** The mass of reactants and the yield of obtained PANI salts.

Sample	Aniline (mL)	*p*-TSA (g)	APS (g)	H_2_O (mL)	Yield (%)
PANI-0	1	1.9	2.2	0	5.0
PANI-0.25	1	1.9	2.2	0.25	7.8
PANI-0.5	1	1.9	2.2	0.5	23.7
PANI-0.75	1	1.9	2.2	0.75	55.0
PANI-1	1	1.9	2.2	1	73.2

### 2.2 Ultraviolet-Visible(UV-Vis) Spectra

**Figure 2:**
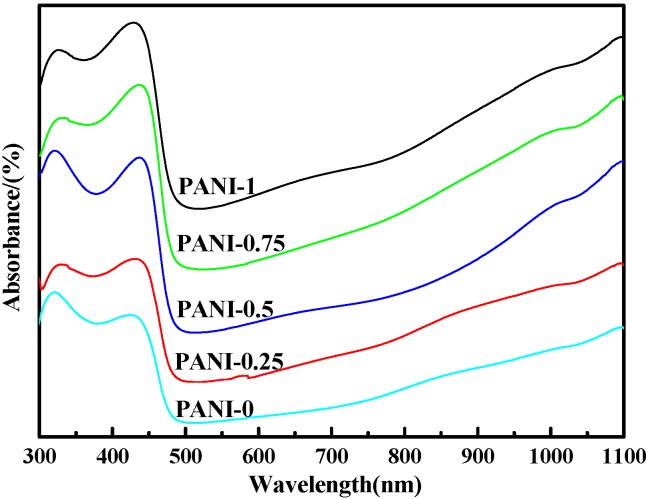
Ultraviolet-Visible (UV-Vis) spectra of PANI with different amounts of water in *m*-cresol.

Figure 2 represents the UV–Vis absorption spectra of PANI salts in *m*-cresol solution. As is clarifiedin Figure 2, all PANI salts show two characteristic absorption peaks at ~315–342 nm and ~405–442 nm. The absorption peak at ~315–342 nm can be ascribed to π-π^*^ transition of the benzenoid rings, while the peak at 405–442 nm can be attributed to polaron-π^*^ transition [18,25]. The peak at ~315–342 nm can also be attributed to the leucoemeraldine (fully reduced form) of PANI, while the peak at ~405–442 nm is due to the protonated form of PANI [18]. However, when an intense free carrier tail commencing at about 1000 nm appears, the appearance of the intense free carrier tail in the near infrared region is taken as an indication for the delocalization of electrons in the polaron band of the “expanded coil,” which is originated from the “compact coil” through the secondary-doping-induced conformational transition by *m*-cresol [34,35]. The reason is that *m*-cresol not only serves as a solvent, but also acts as an efficient secondary dopant [36]. Comparing the peaks position at ~315–342 nm and ~405–442 nm, one can see that the corresponding peak of PANI-0.25 and PANI-0.75 slightly shifts to a higher wavelength, meaning that the conjugate degree of them is larger and have more quinoid ring modes than others. The FTIR studies show that both of them have a higher oxidation degree, and the UV–Vis results are consistent with FTIR.

### 2.3. X-ray Diffraction (XRD) Analysis

**Figure 3:**
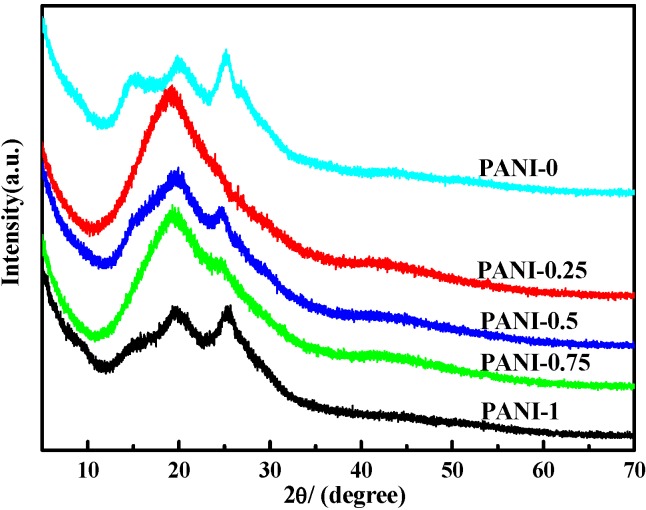
X-Ray Diffraction (XRD) spectra of PANI with different amounts of water.

X-ray diffraction tests can provide a great deal of information on structural aspects. The XRD patterns for the PANI salts are shown in Figure 3. It is noted that the details of the crystal structure depend upon the counterion used. From the XRD, the Bragg diffraction shoulders of 2θ = ~15°, 2θ = ~19.4° and 25° can be found in obtained PANI salts. These peaks manifest emeraldine salt form of PANI [20,37], indacting that PANI salts have some crystallinity. The crystallinity of PANI can be ascribed to the repetition of benzenoid and quinoid rings in PANI chains [38]. The peak centered at 2θ = ~19.4° may be ascribed to a periodicity parallel to the polymer chain, and the peak at 2θ = ~25° may be caused by the periodicity perpendicular to the polymer chain [39,40,41]. The peak at 2θ = ~20° also represents the characteristic distance between the ring planes of benzene rings in adjacent chains or the close-contact inter-chain distance [42]. However, the crystallization property of PANI salts is affected by different amounts of deionized water in solid-state polymerization. As for PANI-0.25 and PANI-0.75, their crystallinity are lower.

### 2.4. Morphology

**Figure 4:**
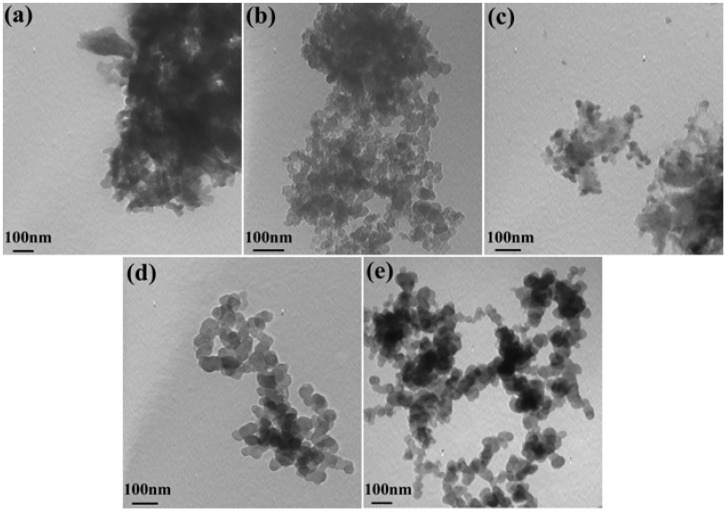
Transmission electron microscopy (TEM) image of PANI with different amounts of water: (**a**) PANI-0; (**b**) PANI-0.25; (**c**) PANI-0.5; (**d**) PANI-0.75; (**e**) PANI-1.

Figure 4 represents the TEM images of PANI salts. It is well known that the morphology of PANI depends on the polymerization method and temperature. It is apparent from these images that different amounts of deionized water effectively control the dispersion and the morphology of particles. As can be seen from the TEM, when adding more water, the morphology of the obtained polyaniline salt is spherical and shows good dispersion. The diameter of all PANI salts is about 60 nm and these particles are at the nanoscale. However, as for PANI-0, PANI-0.25 and PANI-0.5, the particles are irregularly shaped and aggregated. With the addition of water in the beginning of this solid-state synthesis, the obtained PANI salts have better dispersion, as illustrated by PANI-0.75 and PANI-1 in Figure 4 (d) and Figure 4 (e), respectively.

### 2.5. Electrochemical Properties

**Figure 5:**
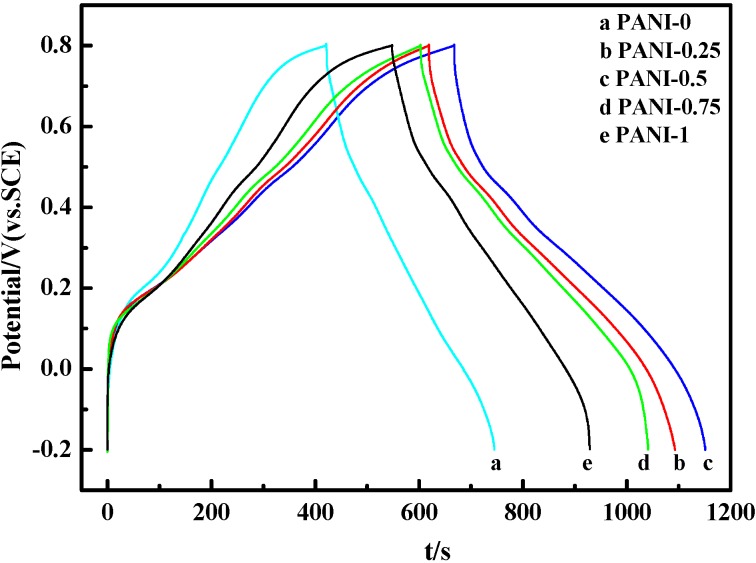
Charge-discharge of PANI salts at the same current density of 3 mA^.^cm^−2^ in 1 M H_2_SO_4_ electrolyte.

To investigate the application of PANI as electrode materials in a supercapacitor, Figure 5 shows the galvanostatic charge-discharge curves of PANI salts at 3 mA^.^cm^−2^ in 1 M H_2_SO_4_. The specific capacitance (SC) of the electrode material is calculated by means of SC = (I × Δt)/(ΔV × m) [43], where I is charge-discharge current, Δt is the discharge time, ΔV is the electrochemical window(1 V), and m is the mass of active materials within the electrode(3 mg). The SC of PANI salts calculated from Figure 5 are as follows: PANI-0: 324 F^.^g^−1^, PANI-0.25: 474 F^.^g^−1^, PANI-0.5: 483 F^.^g^−1^, PANI-0.75: 438 F^.^g^−1^, PANI-1: 381 F^.^g^−1^, respectively. It can be seen that all the charge-discharge curves are not ideal straight lines, indicating the process of a faradic reaction, and the SC comes from pseudocapacitance resulting from the fast reversible oxidation and reduction processes [44]. Comparing these values, the PANI-0.5 shows a higher SC, while PANI-0 and PANI-1 are 324 and 381 F^.^g^−1^, respectively, indicating that the amount of deionized water affects heavily the specific capacitance (SC) of PANI salts in the solid-state reaction. Moreover, PANI-0.5 has the highest specific capacitance value of 483 F^.^g^−1^, which may be related to the formation of polyaniline in its doped half-oxidized emeraldine state.

Due to the higher SC, the possibility of PANI-0.5 as the potential application of capacitor is higher than others. The following work is a detailed study of the electrochemical properties about PANI-0.5.

**Figure 6:**
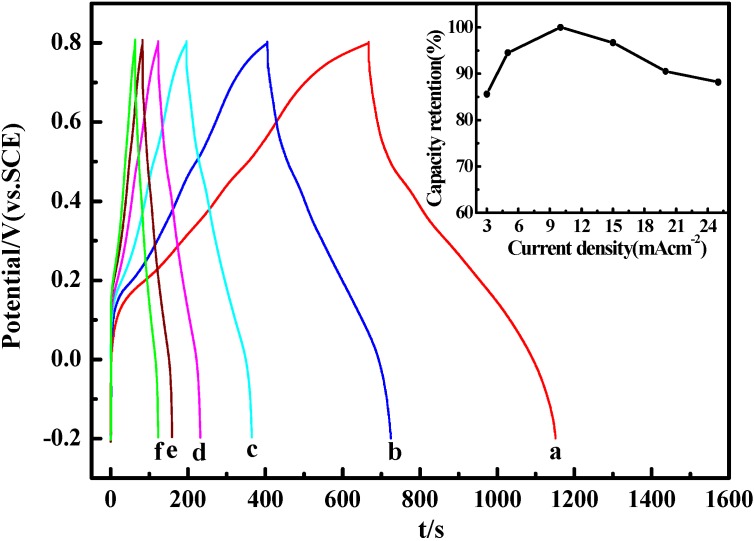
Charge-discharge curves of PANI-0.5 at different current densities in 1 M H_2_SO_4_ electrolyte: (**a**) 3 mA^.^cm^−2^; (**b**) 5 mA^.^cm^−2^; (**c**) 10 mA^.^cm^−2^; (**d**) 15 mA^.^cm^−2^; (**e**) 20 mA^.^cm^−2^; (**f**) 25 mA^.^cm^−2^. The inset shows the capacity retention as a function of current density.

Figure 6 shows the galvanostatic charge-discharge curves of PANI-0.5 at different current densities in 1 M H_2_SO_4_ electrolyte. The SC calculated from Figure 6 are 483, 533.3, 564, 545.5, 510.6, 497.5 F^.^g^−1^ at the current density of 3, 5, 10, 15, 20 and 25 mA^.^cm^−2^, respectively. The variations in the capacity retention as a function of the current density are plotted in the inserted image. The charge-discharge curves exhibit mirror-like images, indicating a reversible oxidation process and better electrochemical capacitance performance. From the illustration in Figure 6, when the current density is 10 mA^.^cm^−2^, the SC reaches the maximum value of 564 F^.^g^−1^. The corresponding capacitance retention ratio still reaches *circa* 90% with growth of current densities from 3 to 25 mA^.^cm^−2^, indicating PANI-0.5 as electrode material can be maintained under very high power operations. However, the SC increases with increasing of charge–discharge current densities from 3 mA cm^−2^ to 10 mA cm^−2^, this is similar to the earlier report [45], in which the PANI nanowire arrayed electrodes was synthesized by means of anodic deposition technique. According to the previous report, this increase of the SC may be attributed to the existence of various forms of pores and pore diameters in the electrode that resulted from the different nanosized structure, and it seems that some pores with small diameter can be invaded by ions from the electrolyte with high charging current [46]. As it is noted in Figure 6, the SC gradually decreases after the increase of current density from 10 mA cm^−2^ to 25 mA cm^−2^, and it is in accordance with the common belief that the SC decreases with the increasing of current density [47]. The reason for this is that the electrolyte ion cannot penetrate well into the inner of active materials due to slow diffusion at large current density.

**Figure 7:**
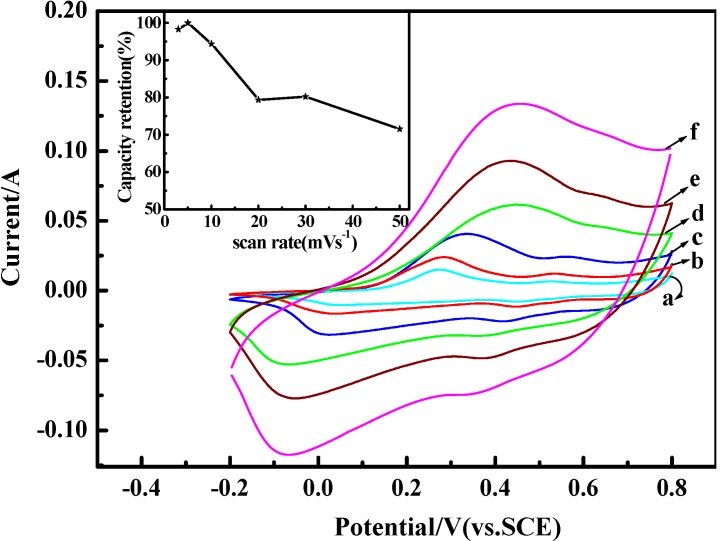
Cyclic voltammetry curves of PANI-0.5 at different scan rates: (**a**) 3 mV^.^s^−1^; (**b**) 5mV^.^s^−1^; (**c**) 10 mV^.^s^−1^; (**d**) 20 mV^.^s^−1^; (**e**) 30 mV^.^s^−1^; (**f**) 50 mV^.^s^−1^. The electrolyte is 1 M H_2_SO_4_; the inset shows the capacity retention as a function of scan rate.

Figure 7 gives the CV curves of PANI-0.5 as electrode materials measured from −0.2 to 0.8 V at different scan rates in 1 M H_2_SO_4_ electrolyte. As shown in Figure 7, the peak currents rapidly increase and the redox peaks become broader with the increase of the scan rate from 3 to 50 mV^.^s^−1^. The oxidation peaks shift positively and the reduction peaks shift negatively with the increase of the scan rate. The gradual increase of the current as a function of the scan rate means a good rate capability of PANI-0.5, and the shifts of the redox peaks are due to the resistance of the electrode. Previous studies show that the SC of the electrode can also be estimated from the CV curves, and the formula is $C=\int_{E1}^{E2}i(E)dE/2vm(E2-E1)$ [48], where C is the specific capacitance (SC) of the individual sample. E_1_, E_2_ are the cutoff potentials in cyclic voltammetry. i(E) is the instantaneous current. $\int_{E1}^{E2}i(E)dE$ is the total voltammetric charge obtained by the integration of a positive and negative sweep in cyclic voltammograms. (E_2_ − E_1_) is the potential window width, and m is the mass of the individual sample. The SC calculated from Figure 7 are 583.3, 593.3, 560, 471, 476, 424.6 F^.^g^−1^ at the scan rate of 3, 5, 10, 20, 30 and 50 mV^.^s^−1^, respectively. However, it should be noted that the SC calculated from CV are different from those derived from galvanostatic charge–discharge test, which is mainly due to the different testing systems applied. The variation in the SC of the electrodes as a function of the scan rate is also plotted in the inserted image, and the capacitance retention ratio can still reach about 72% even at the scan rate of 50 mV^.^s^−1^.

**Figure 8:**
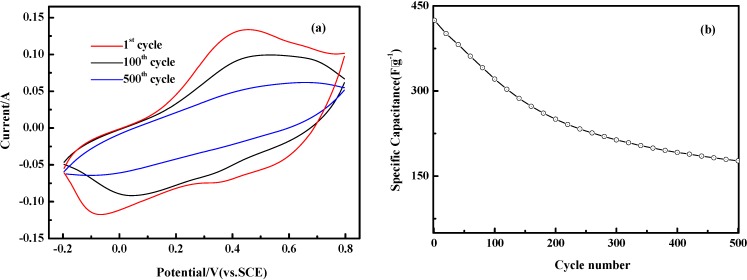
(**a**) CV curves for the PANI-0.5 electrode for the 1st, 100th and 500th cycle from stability studies using 1 M H_2_SO_4_ electrolyte solution at 50 mV^.^s^−1^; (**b**) Variation in the specific capacitance as a function of the number of cycles.

It is well known that long-term cycle stability is one of the most important factors to consider for conducting polymers in supercapacitor applications. Figure 8 (a) shows the CVs for the 1st, 100th and 500th cycle at 50 mV^.^s^−1^ for PANI-0.5 electrode from our stability studies. The variation of specific capacitance with cycle number is presented in Figure 8 (b) and indicates that the specific capacitance of the PANI-0.5 as supercapacitor decays at a relatively slow rate. After 500 cycles, the capacitance retention ratio still reaches about 42%, indicating high cycling stability of the nanostructured polyaniline electrode. Any observed net loss in CV areas from Figure 8a could be attributed to loss of active material through partial dissolution of the materials. Conductive polymer-supercapacitor electrodes often suffer from cycle degradation issues caused by mechanical problems, such as swelling and shrinking, during the doping–dedoping process [3].

**Figure 9:**
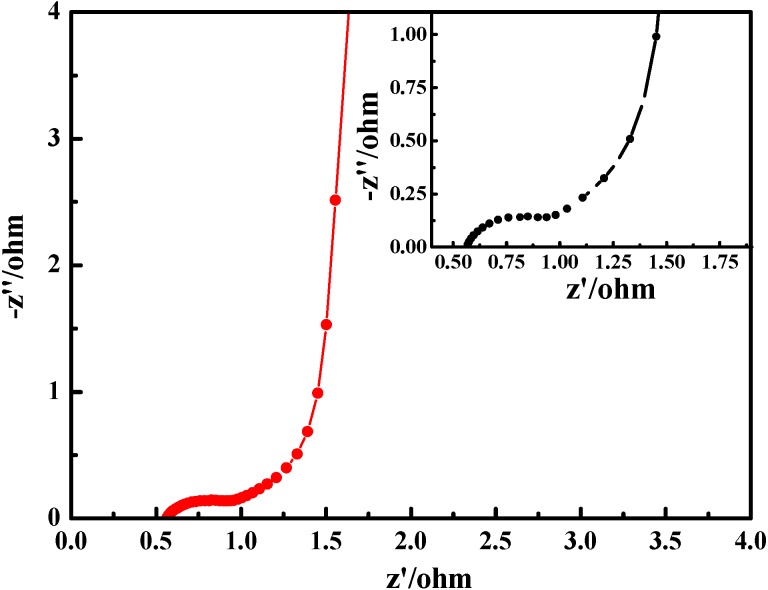
Electrochemical impedance spectroscopy of PANI-0.5 at open-circuit potential with ac-voltage amplitude of 5 mV over the frequency range of 10^−2^–10^5^ Hz in 1 M H_2_SO_4_ electrolyte；the inset shows the enlargement of a part of Electrochemical impedance spectroscopy.

Electrochemical impedance spectroscopy (EIS) has been widely used to study the redox processes of electrically conducting polymers. It can be seen from Figure 9 that the EIS plot contains two well-separated patterns. To see clearly the cross point of a semicircle with the real axis and the radius of the semicircular, the enlargement of a part of Electrochemical impedance spectroscopy (EIS) is shown in the inset of Figure 9. First, the high frequency intercept of the semicircle with the real axis can be used to evaluate the value of internal resistance, which includes the resistance of the electrolyte solution, the intrinsic resistance of the active material and the contact resistance at the interface active material/current collector. Its value is approximately 0.57 Ω, while the radius of the semicircular is 0.22 Ω. Second, the imaginary impedance at low frequency reveals a slightly tilted vertical line of a limiting diffusion process, which is a characteristic feature of pure capacitive behavior [49]. These results also further illustrate that PANI-0.5 can be used as electrode material for supercapacitors.

## 3. Experimental Section

### 3.1. Materials

Aniline (99.5%) and ammonium peroxydisulfate ((NH_4_)_2_S_2_O_8_, APS) of analytical-reagent grade were purchased from Xi’an Chemical Reagent Company (China), *p*-toluenesulphonic acid (*p*-TSA) was obtained from Acros Organics (Shanghai Aladdin Reagent Company, China). Aniline was purified by double distillation under reduced pressure prior to use and stored in the refrigerator; all other chemicals and solvents were used as received without further purification. Deionized water was used throughout.

### 3.2. Synthesis of Polyaniline Salts

A typical solid-state synthesis of polyaniline doped with *p*-toluenesulphonic acid (*p*-TSA) was as follows: 1 mL of aniline and 1.9 g *p*-TSA were grinded to mix each other in the mortar, and then 0.5 mL of deionized water was added. After grinding, the mixture became a white paste and 2.2 g of APS was added by further grinding for 30 min. Finally, the color of the powder changed to dark green. The dark green powder was washed with ethylether, ethanol and deionized water repeatedly until the filtrate was colorless, then the powder was dried under vacuum at 50 °C for 48 hr. The sample is designated as PANI-0.5. Using the same method, the other samples were synthesized just by changing the amount of deionized water from 0 to 1 mL. The specific reactants mass, the amount of adding water and the yield of obtained PANI salts were listed in Table 1.

### 3.3. Structure Characterization

The FTIR spectra of the samples were measured on a BRUKERQEUINOX-55 fourier transform infrared spectrometer (Billerica, MA, USA) at a resolution of 4 cm^−1^ using the KBr technique. UV-Vis spectra of the samples were recorded on a UV-Visible spectrophotometer (UV4802, Unico, USA). XRD patterns have been obtained by using a Bruker AXS D8 diffractometer and the scan range (2θ) was 5°–70°, with monochromatic Cu-Ka radiation source (λ = 0.15418 nm). Transmission electron microscopy (TEM) experiments were performed on a Hitachi 2600 electron microscope. The samples for TEM measurements were prepared by placing a few drops of products ethanol suspension on copper supports.

### 3.4. Electrochemical Tests

The working electrode was prepared by mixing 85 wt.% active materials (3 mg), 10 wt.% carbon black and 5 wt.% polytetrafluoroethylene (PTFE) to form slurry. The slurry was coated onto a graphite current collector (area: 1 cm^2^), then dried at 60 °C for 24 hr under vacuum. The electrochemical measurements, including galvanostatic charge-discharge, cyclic voltammetry (CV), cycle life and electrochemical impedance spectroscopy (EIS) techniques were carried out in a three-electrode glass cell: a standard calomel reference electrode (SCE), a platinum counter electrode, and the working electrode. Galvanostatic charge–discharge tests, cyclic voltammetry and cycle life were performed in the potential window ranging from −0.2 V to 0.8 V and conducted at different scan rates and different current densities using CHI660C electrochemical working station. EIS measurements were performed at open-circuit potential by using Zennium 40084. Data were collected in the frequency range of 10^−2^ Hz to 10^5^ Hz. The electrolytes were 1 M H_2_SO_4_ solution in all the electrochemical tests.

## 4. Conclusions

In this work, a series of PANI doped with *p*-TSA were synthesized by solid-state polymerization. The results showed that different amounts of deionized water had a great influence on the structure of PANI, such as the oxidation degree, conjugate level, crystallinity, yield as well as morphology. Comparison results showed that the small amount of water can affect the amount of H^+^ in the reaction system, and this would in turn bring different oxidation degree, conjugate level and crystallinity of PANI. With the presence of more water, the reaction medium would be more acidic, and the inter-diffusion rate of the reactants can be accelerated. The more acidic condition could bring the reduced PANI, while the higher inter-diffusion rate of the reactants could be a benefit for the formation of oxidized PANI. As the results of the two opposite effects, the more reduced PANI occurred in the case of 0 mL and 1 mL of water, while the more oxidized PANI occurred in the case of 0.25 mL and 0.75 mL of water. Therefore, the half-oxidized emeraldine phase of PANI occurred only in the case of 0.5 mL of water, and, consequently, the PANI displayed better electrochemical performances than others.
